# Protection against Recurrent Stroke with Resveratrol: Endothelial Protection

**DOI:** 10.1371/journal.pone.0047792

**Published:** 2012-10-17

**Authors:** Darren Clark, Ursula I. Tuor, Roger Thompson, Adam Institoris, Angela Kulynych, Xu Zhang, David W. Kinniburgh, Ferenc Bari, David W. Busija, Philip A. Barber

**Affiliations:** 1 Departments of Physiology and Pharmacology, Clinical Neurosciences and Radiology, University of Calgary, Calgary, Canada; 2 Hotchkiss Brain Institute and Faculty of Medicine, University of Calgary, Calgary, Canada; 3 Department of Medical Physics and Informatics, University of Szeged, Szeged, Hungary; 4 Department of Physiology, University of Szeged, Szeged, Hungary; 5 Department of Pharmacology, Tulane University, New Orleans, Louisiana, United States of America; 6 Alberta Centre for Toxicology, University of Calgary, Calgary, Canada; University Medical Center Utrecht, The Netherlands

## Abstract

Despite increased risk of a recurrent stroke following a minor stroke, information is minimal regarding the interaction between injurious mild cerebral ischemic episodes and the possible treatments which might be effective. The aim of the current study was to investigate recurrent ischemic stroke and whether resveratrol, a nutritive polyphenol with promising cardio- and neuro- protective properties, could ameliorate the associated brain damage. Experiments in adult rats demonstrated that a mild ischemic stroke followed by a second mild cerebral ischemia exacerbated brain damage, and, daily oral resveratrol treatment after the first ischemic insult reduced ischemic cell death with the recurrent insult (P<0.002). Further investigation demonstrated reduction of both inflammatory changes and markers of oxidative stress in resveratrol treated animals. The protection observed with resveratrol treatment could not be explained by systemic effects of resveratrol treatment including effects either on blood pressure or body temperature measured telemetrically. Investigation of resveratrol effects on the blood-brain barrier *in vivo* demonstrated that resveratrol treatment reduced blood-brain barrier disruption and edema following recurrent stroke without affecting regional cerebral blood flow. Investigation of the mechanism in primary cell culture studies demonstrated that resveratrol treatment significantly protected endothelial cells against an in vitro ‘ischemia’ resulting in improved viability against oxygen and glucose deprivation (39.6±6.6% and 81.3±9.5% in vehicle and resveratrol treated cells, respectively). An inhibition of nitric oxide synthesis did not prevent the improved cell viability following oxygen glucose deprivation but SIRT-1 inhibition with sirtinol partially blocked the protection (P<0.001) suggesting endothelial protection is to some extent SIRT-1 dependent. Collectively, the results support that oral resveratrol treatment provides a low risk strategy to protect the brain from enhanced damage produced by recurrent stroke which is mediated in part by a protective effect of resveratrol on the endothelium of the cerebrovasculature.

## Introduction

Thrombo/embolic ischemic stroke is a leading cause of death and long-term neurological disability. An important population of patients at risk of stroke is one that has had a transient ischemic attack (TIA) or minor stroke. In these patients cerebral vessel occlusion is of short duration and its recannalization is followed by near or complete functional recuperation. Despite functional recovery, there is limited knowledge concerning the extent of full tissue recovery. There is increasing evidence that there can be ischemic damage not always detectable with standard diagnostic imaging techniques. Health status of the brain is likely crucial in these patients considering they are at higher risk of a recurrent stroke, with stroke recurrence being reported in 6–15% of patients within 90 days and in up to 21% of patients by one year post initial insult [1], [2]. The degree of cerebral recovery can likely affect the brain's susceptibility to a second ischemia. Indeed, we have observed this in our novel model of recurrent stroke in the rat which incorporates a transient middle cerebral artery occlusion (MCAO) (e.g. a TIA or mild stroke) followed by a second occlusion with recannalization [3]. We found that an MCAO of short duration (mild ischemic stroke) can result in scattered necrosis not well detected with standard MRI and such an insult followed by a subsequent moderate stroke had exacerbated damage [3]. This was different from the preconditioning protection observed with a mild ischemic insult that does not produce permanent damage [4] and little is known about the interaction of multiple insults causing mild damage or minor stroke. Also unknown is the potential effectiveness of treatments administered after an initial insult and prior to a recurrent insult. The dietary polyphenol resveratrol (3,4′,5-trihydroxystilbene), found naturally in foods such as berries, nuts, and grape skins has been considered a highly promising treatment for recurrent stroke in light of its documented range of beneficial cardiovascular and neurological benefits [5]–[11] with minimal side effects [12].

Thus, in the present study we used a novel model of recurrent stroke in the rat to investigate the interaction between multiple mild ischemic insults and the effects of resveratrol treatment. The results demonstrate that brain damage is enhanced when a mild recurrent ischemic stroke follows an initial mild ischemic insult. Oral administration of resveratrol between insults was found to reduce ischemic cell death. The beneficial effects of resveratrol treatment were not related to altered regulation of systemic blood pressure, body temperature or regional cerebral blood flow (CBF) but are consistent with it acting, at least partially, via beneficial effects on endothelial function.

## Results

### Significant Levels of Resveratrol in Blood and Tissues Following Oral Treatment

**Figure 1:**
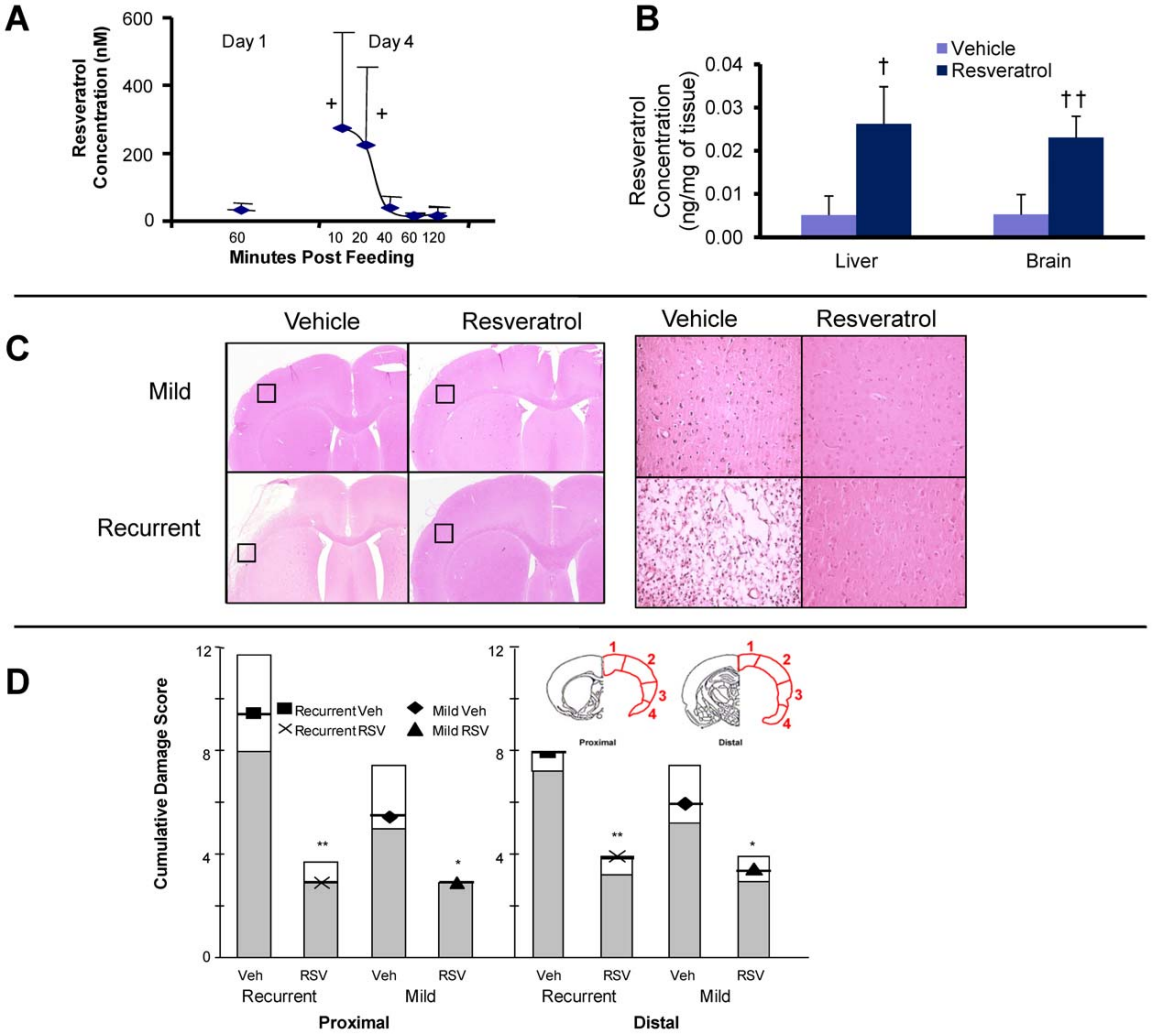
Mean (±SD) amounts of resveratrol (RSV) detected in the plasma (A), and liver and brain tissue (B), of naive animals treated with daily oral doses of RSV (25 mg/kg). Hematoxylin and eosin stained sections (C) from scanned sections (left) and magnified insets (20x; right) depict scattered cell death following a single mild stroke and extensive cell death following a recurrent stroke. Less cell death is observed with RSV treatment. Quantitative assessment of damage as a cumulative injury score (D) demonstrated that there was reduced cell death in the RSV treated groups compared with the respective vehicle (Veh) treated group in either proximal or distal cerebral cortex. Data presented as median (+/− first and third quartile). Different from respective vehicle (Veh): +P<0.05 (ANOVA and Dunn's Test), †P<0.05 or ††P<0.006 (Students t-test), *P<0.03 or **P<0.002 (ANOVA on ranks and Mann-Whitney Rank Sum Test).

Experiments were performed to investigate the concentrations of resveratrol in blood and tissues following oral resveratrol administration. Significant levels of resveratrol were detected using high performance liquid chromatography tandem mass spectrometry (LC/MS/MS) in the plasma and tissues of resveratrol treated animals; levels reaching a peak that fell quickly soon after feeding (Fig. 1 A). Resveratrol was detected in significant amounts in liver and brain homogenates of treated rats following daily doses (25 mg/kg/day) for four doses (Fig. 1 B). Small amounts of resveratrol were also detected in the tissue of control animals, consistent with the modest levels of resveratrol present in the regular lab rat diet.

### Enhancement of Cell Death by Recurrent Stroke and its Reduction by Resveratrol

Of the 42 animals randomized to MCAO, six died as a result of surgical complications. During MCAO all animals had >90% reductions in cortical perfusion as measured in the core region using a single laser Doppler probe. A mild ischemic stroke produced cortical injury and scattered cell necrosis evident seven days post-insult (Fig 1 C). The damage observed following recurrent mild stroke was more severe often consisting of regions of infarct and this was reflected in a higher damage score (P<0.05) (Fig.1 D).

Oral treatment with resveratrol for three days prior to a single mild stroke, reduced the extent of damage observed compared with vehicle treated animals (Fig 1 C, D). Similarly, in resveratrol treated animals that were administered resveratrol immediately following the initial insult and for three more doses on each of the next three days, there was a reduced damage score and enhanced protective effect compared to vehicle treated animals (Fig. 1 D).

### Lack of Resveratrol Mediated Alterations in Physiological Variables that Potentially Affect Stroke Outcome

The effects of resveratrol on physiological parameters potentially affecting ischemic stroke outcome were investigated carefully. During MCAO, blood glucose, mean arterial blood pressure, PO2, PCO2, PH, and tympanic temperature were similar between groups (Table 1). All rats recovered well from both single and recurrent stroke procedures with a similar gain in weight in resveratrol and vehicle treated animals. At the time of MCAO, laser Doppler flow measured within the core (proximal parietal cortex) ipsilateral to the occlusion decreased to: 8.0±1.8% and 8.6±2.7% of baseline in the vehicle and resveratrol treated single mild stroke groups; 8.2±3.2% and 6.4±2.6% in the vehicle and resveratrol treated recurrent stroke groups (first mild stroke); 9.4±4.2% and 8.6±1.1% in the vehicle and resveratrol treated recurrent stroke groups (second mild stroke), respectively. No significant differences were found in the flows detected either in the proximal or distal cortical regions between resveratrol and vehicle treated groups at either onset or end of ischemia. Five minutes following removal of all three clips on the arteries, blood flow in the ipsilateral parietal cortex proximally returned to greater than pre-occlusion levels (range of 114 to 141%) and were not different between groups (data not shown). Similar responses were observed distally (means of 128.5, 132.2, 116.8 and 114.6% of initial flow in the recurrent vehicle, recurrent resveratrol, single vehicle and single resveratrol groups, respectively; data not shown).

**Table 1:** Mean+ body temperature and arterial blood gas values during and following either a single mild stroke or a first and second recurrent stroke.

Stroke Type	Treatment	Temperature (°C)		PO2 (mm Hg)		PCO2 (mm Hg)		pH	
		During	Reperf	During	Reperf	During	Reperf	During	Reperf
Mild Stroke	Veh	36.4	36.6	102	101	49.5	45.3	7.31	7.34
		±0.3	±0.2	±7	±23	±14	±15	±0.08	±0.09
Mild Stroke	RSV	36.5	36.8	128	111	36.2	39.4	7.4	7.37
		±0.2	±0.4	±38	±2	±4	±7	±0.02	±0.03
Recurrent 1^st^	Veh	36.5	36.7	110	109	42.7	43.8	7.36	7.33
		±0.4	±0.4	±11	±20	±3	±3	±0.05	±0.06
Recurrent 2^nd^	Veh	36.4	36.7	110	102	40.6	50.9	7.37	7.33
		±0.4	±0.5	±12	±26	±9	±6	±0.05	±0.01
Recurrent 1^st^	RSV	36.4	36.6	118	123	38	38	7.41	7.37
		±0.3	±0.3	±25	±27	±5	±2	±0.04	±0.03
Recurrent 2^nd^	RSV	36.4	36.6	117	124	37.6	42.4	7.41	7.39
		±0.3	±0.3	±20	±18	±5	±12	±0.04	±0.06

+ data presented as mean±SD (n = 3-6), n.s. Mean hematocrits – 35-40. Abbreviations: Reperf – Reperfusion, Veh- Vehicle, RSV – resveratrol.

**Figure 2:**
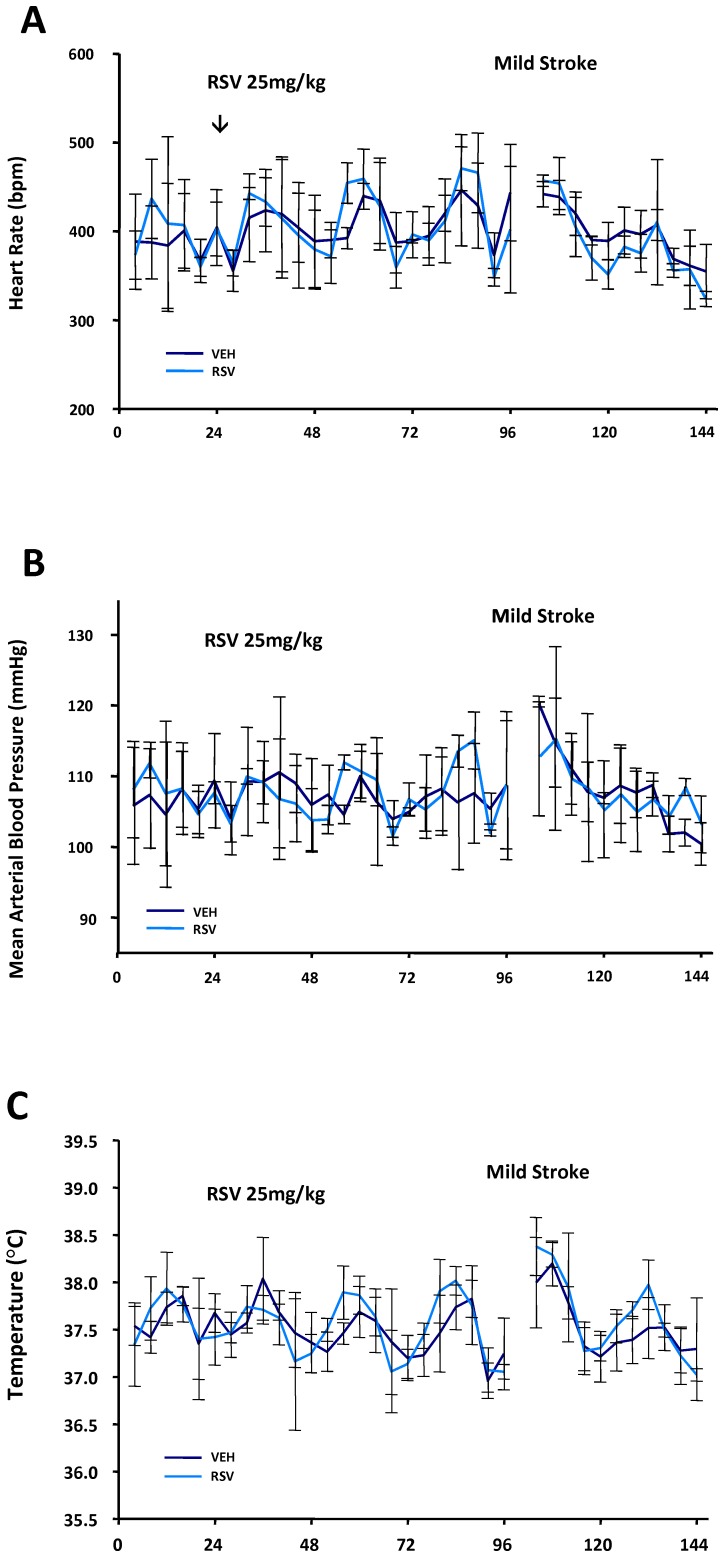
Telemetric measures of heart rate (A), mean arterial blood pressure (B) and body temperature (C). Mean (±SD) values were similar in resveratrol (RSV, 25 mg/kg/day for 4 doses) and vehicle (Veh) treated rats at all times for 24 hr prior to treatment, for 3 days during treatment (prior to a mild stroke) and during the 48 hr of monitoring thereafter.

Despite a lack of evidence for acute physiological differences during MCAO in the anesthetized animals, it was possible that resveratrol had systemic effects which could be more readily detected in conscious animals. There has been evidence suggesting that resveratrol can affect blood pressure [6], [13], [14] or body temperature [15], although such findings have not been consistent [16]. Thus, in additional groups of unanesthetized animals, we further investigated the systemic circulatory and temperature control effects of daily resveratrol administration using implanted telemetry probes. In these animals, resveratrol treatment did not alter telemetrically measured heart rate, mean arterial blood pressure or core temperature at any time points prior to or following resveratrol treatment compared with vehicle treated controls (Fig. 2 A–C).

### Reduction of Markers of Inflammatory Changes and Nitroxidative Stress with Resveratrol Treatment

Next we considered whether resveratrol had beneficial effects on signs of inflammation or oxidative stress following mild or recurrent ischemic stroke. Using immunohistochemistry, we assessed alterations in staining for reactive astrocytes using anti-glial fibrillary acidic protein (GFAP), alterations in staining for microglia/macrophage reactivity using anti-ED1, and altered oxidative stress using immunostaining with anti-nitrotyrosine (NT), a product of free radical oxidation of nitric oxide [17], [18].

**Figure 3:**
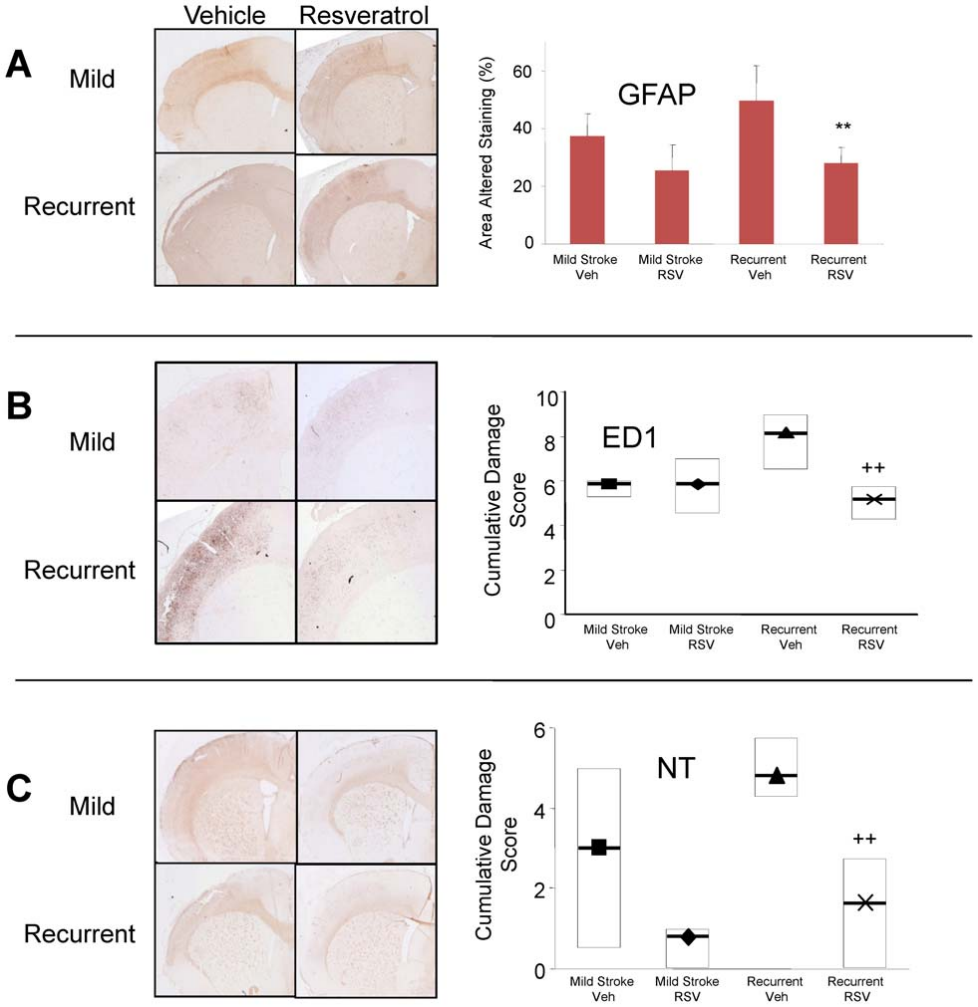
Representative brain sections depicting resveratrol (RSV) effects on glial activation, microglial/macrophage activation and nitrosative stress using respective immunostaining for: A - glial fibrillary acidic protein (GFAP), B – ED1 and C - nitrotyrosine (NT). Staining was assessed as the mean (±SD) area of altered GFAP stain presented as a percentage of the contralateral hemisphere or according to a median cumulative damage score (+/− first and third quartiles) of altered NT and ED1 staining. Significant RSV and vehicle (Veh) treatment differences were detected between respective stroke groups. ** P<0.005 (ANOVA and Student-Newman Keuls Test), ++ p<0.005 (ANOVA and Mann-Whitney Rank Sum Test).

Following a mild ischemic stroke, sections immunostained with GFAP demonstrated areas of increased astrogliosis in peri-infarct/penumbral regions- an effect which was enhanced with recurrent stroke (Fig. 3 A). The group of animals treated with resveratrol between their initial and recurrent stroke demonstrated significant reductions in areas of altered astrocytic GFAP staining compared with the vehicle treated group (P<0.001).

Following a mild ischemic stroke, there was increased ED1 staining of microglia/macrophages evident within the ischemic core and peri-infarct/penumbral regions (Fig 3 B). Quantitative assessment demonstrated that in animals with recurrent stroke there was a reduced cumulative staining score for ED1 compared with vehicle treated animals (P<0.008) suggestive of a reduced inflammatory response.

Similar to ED1, NT staining was increased ipsilaterally in the ischemic region following either a mild or recurrent ischemic stroke (Fig 3 C). In resveratrol treated animals with recurrent stroke there was a significant decrease in staining for NT in the ischemic cortex ipsilaterally (P<0.009) suggestive of a reduced production of nitroxyl radicals in the brains of resveratrol treated animals with recurrent stroke.

### Resveratrol Does Not Alter Local Regional CBF During or After Recurrent Stroke but does improve BBB function

**Figure 4:**
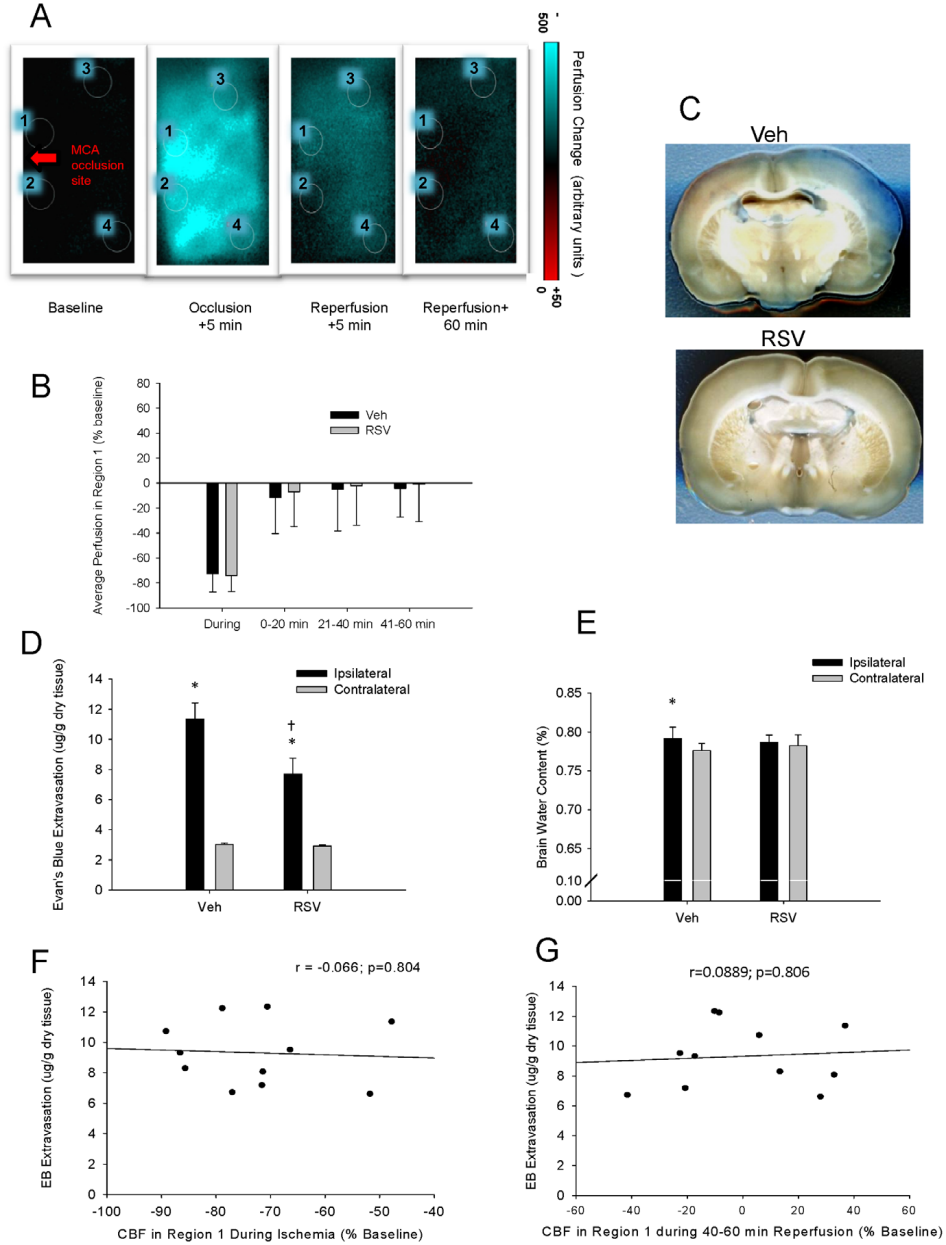
Representative color-transformed laser speckle difference images (A) showing regional perfusion changes relative to baseline values for an animal during recurrent mild stroke that was produced three days following an initial mild stroke. Perfusion values were assessed in four different regions (1 to 4) for each animal according to positions relative to the site of middle cerebral artery occlusion (arrow). Mean perfusion values were similar in resveratrol (RSV) and Vehicle (Veh) treated rats for all regions at all time points with mean values for Region 1 shown in Panel B. In the same animals, blood brain barrier function was assessed with Evans Blue and (C) shows representative coronal slices of brain from rats treated with resveratrol (RSV) and Vehicle (Veh) showing greater Evans Blue extravasation indicative of BBB dysfunction in Veh treated rats. The amount of Evans Blue present in the cortex was quantified using fluorescent spectrometry (E) and RSV-treated rats had significantly less Evans Blue extravasation in the ipsilateral cortex compared with Veh-treated rats. Resveratrol treatment also prevented edema, as only Veh-treated rats showed a significant increase in cortical brain water weight (F) on the injured side two days post-stroke. The extent of Evans blue extravasation was not correlated to the regional perfusion changes (e.g. in Region 1), either during recurrent stroke (G) or after recurrent stroke (H), indicating that blood-brain barrier disruption did not depend on acute flow differences between groups. * p<0.05, different from contralateral (Student's paired t-test). † p<0.001, different from Veh (ANOVA with a Student-Newman Keuls test).

The mechanisms causing stroke injury are complex and maintenance or restoration of tissue perfusion and vascular integrity may be key for mitigating complex neuroinflammatory processes at the blood brain barrier precluding neuronal cell death [19], [20]. Various reports on vascular effects of resveratrol support the possibility of enhanced vascular reactivity including improved regional CBF or improved BBB integrity [7], [10], [21]. Thus we performed additional experiments that investigated the effect of resveratrol treatment on regional blood flow during and following recurrent stroke measured with laser speckle imaging. Although regional CBF was significantly reduced from baseline during ischemia in all defined regions of interest (Regions 1–4; P<0.001) and perfusion values differed between the different regions examined, there were no significant differences between resveratrol and vehicle treated rats at any time point, in any region (Fig. 4 A, B).

In contrast to a lack of effect of resveratrol on CBF, there were differences in BBB function in these same animals. Recurrent mild stroke resulted in an increase in Evans Blue extravasation in the cortex ipsilateral to the injury that was more marked in vehicle compared to resveratrol treated rats (Fig. 4 C). Resveratrol treatment significantly reduced the amount of Evans blue leakage through the BBB (P<0.001; Fig. 4 D). Consistent with the changes in vascular permeability, there were corresponding changes in brain water content (Fig 4 E). Brain water content in the ipsilateral compared with the contralateral cortex was significantly increased in vehicle treated rats (P<0.04); whereas, resveratrol treated rats did not have a significant increase in brain water content ipsilaterally at 48 hours following the second stroke. Linear regression between the average flow in Region 1 (the region within the window considered closest to the core) and the amount of Evans Blue present in the tissue after 48 hours, showed that the amount of BBB disruption was not predicted by either the blood flow during ischemia (Fig 4 F, r = −0.066; P = 0.804) or after 40 minutes of reperfusion (20–60 minutes of reperfusion, Fig 4 G, r = 0.0889; P = 0.806). This suggests that the protective effect of resveratrol treatment on the BBB is independent of the original severity of ischemia and the magnitude of acute local reflow.

### Resveratrol Protects Endothelial Cells against Oxygen Glucose Deprivation Cell Death

**Figure 5:**
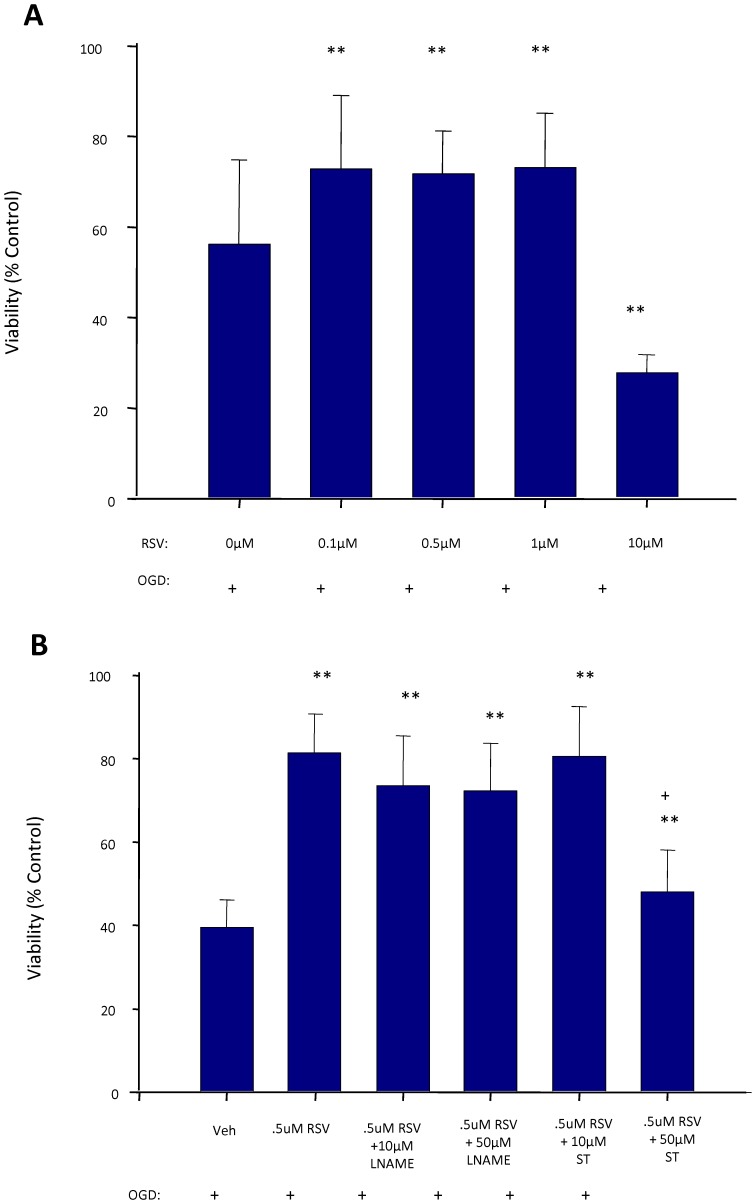
Effect of resveratrol (RSV) treatment on cell viability of endothelial cell cultures exposed to oxygen-glucose deprivation (OGD). Improved viability was observed in endothelial cells treated with RSV at low doses (A). A sirtuin-1 antagonist (sirtinol, ST), but not the nitric oxide synthase inhibitor (L-NAME), partially inhibited the RSV protection in endothelial cells (B). * P<0.05; ** P<0.005 different from 0 µM or vehicle (Veh). + P<0.001 different from 0.5 µM RSV alone. (ANOVA with a Student-Newman Keuls test).

The BBB is localized to cerebral endothelial cells in the cerebral capillaries forming a lumen with tight junctions and specialized transporters. Thus we next investigated whether resveratrol's effects could be studied directly in endothelial cells *in vitro*. Resveratrol treatment of endothelial cells at low doses (0.1–1 µM) for 3 days prior to exposure to oxygen glucose deprivation increased cell viability (Fig.5 A, p<0.001) whereas higher doses (>1 µM) were associated with reduced cell viability. To examine potential pathways involved, the non-specific NOS blocker, (N_ω_-Nitro-L-arginine methyl ester hydrochloride [L-NAME], 10 µM or 50 µM), was investigated and found to not prevent the improved cell viability following oxygen glucose deprivation. However, the SIRT-1 blocker, sirtinol, partially blocked the protection at a dose of 50 µM (P<0.001) (Fig. 5 B). This suggested endothelial protection is, at least in part, SIRT-1 dependent.

## Discussion

The major original finding of the current study is that resveratrol treatment protects against brain injury in our new model of mild recurrent stroke. In addition to a reduction in brain injury, resveratrol treatment lessens oxidative stress and inflammation, prevents edema, protects endothelial cells and preserves BBB function following recurrent stroke, but does not alter regional CBF or systemic variables such as blood pressure and core temperature. The results support that a treatment such as resveratrol is promising for its ability to reduce the severity of damage due to stroke recurrence and could have a significant impact on quality of life and associated costs of caring for stroke survivors.

We show for the first time protection with resveratrol against ischemic brain injury due to recurrent mild stroke. We also observed protection with pre-treatment prior to a single mild stroke which is consistent with what has been found with resveratrol pre-treatment in other stroke models [5], [11], [22]. Resveratrol treatment between mild ischemic strokes also reduced signs of activated astrocytes, reactive nitrogen species, and microglia/macrophage activation suggesting that resveratrol interferes with oxidative and/or inflammatory processes in recurrent stroke, although these effects could be secondary to upstream actions such as activation of SIRT1 deacetylase. This limitation could be addressed in future studies examining more directly the role of these and additional markers of inflammation and oxidative stress. Rather unexpected was our finding that resveratrol-treated rats subjected to recurrent stroke had less ischemic injury than untreated rats with a single mild ischemic stroke. As resveratrol treatment did not begin until one hour after reperfusion from the first stroke, our results indicate beneficial post-treatment effects of resveratrol as well as a pre-treatment effect. Post-ischemic administration of RSV has not been as extensively studied but there is some evidence for protection with post-ischemic administration [9], [23]. Resveratrol having beneficial pre-treatment and post-treatment effects likely contributes to its significant effectiveness in the recurrent stroke model and increases its potential for providing a robust treatment in stroke patients.

Resveratrol has been widely studied but its mechanisms of action are still controversial and poorly defined, including identification of the potential cardiovascular or systemic effects that may modify cerebral ischemic injury or recovery. Determining cardiovascular impact is essential for a better understanding of resveratrol's mode of protection because even minor stroke may lead to effects such as impaired cerebral autoregulation to blood pressure changes [24]. There is evidence in mice that resveratrol treatment has little effect on heart rate [16]. The present study, using continuous telemetric measures in conscious animals, confirmed a lack of effect of resveratrol treatment on heart rate under baseline conditions prior to stroke but also during and post cerebral ischemia. Furthermore, we demonstrated that resveratrol treatment has no appreciable effect on blood pressure before, during, or after a mild ischemic stroke. Body temperature is another critical factor influencing stroke outcome with ischemic brain injury being exquisitely sensitive to even small changes in temperature [25]–[27]. A small (<0.5°C) increase in body temperature has been observed in mice in the first 3 of 7 days of resveratrol treatment [16]. We detected no alterations in body temperature in rats treated with resveratrol before, during or after a mild ischemic stroke. Taken together, the overall lack of cardiovascular and systemic (e.g. temperature) effects of resveratrol both during ischemia and reperfusion suggest that cerebral rather than peripheral changes underlie the protection afforded by resveratrol in young rats.

In addition to the parenchymal protection observed, resveratrol was investigated for its potential to improve the health of the cerebral microvasculature during and after stroke. Microvessel health is essential to neuronal survival and requires preservation of vessel reactivity, adequate cerebral perfusion and optimal BBB function and its associated neurovascular exchange. We found no evidence for resveratrol-mediated augmentation of vascular responsiveness and flow in the ischemic area, during or after ischemic stroke using focal measures of laser Doppler flowmetry or measures of regional CBF with laser speckle imaging. However, we did demonstrate a substantial reduction in BBB disruption and a suppression of edema following recurrent stroke in resveratrol-treated rats that was independent of flow changes.

Several mechanisms may be involved in the protection of the BBB. BBB dysfunction following ischemia is associated with changes in tight junction proteins such as occludin and claudin involving mediators such as matrix metalloproteinases (MMPs) that cause degradation of these tight junction proteins[28], [29]. Studies indicate that resveratrol inhibits increases in MMP expression following oxygen glucose deprivation in vitro or cerebral ischemia in-vivo[30], [31]. Another study has reported protection by resveratrol of the damage to microtubule cytoskeleton and tight junctions in cultured cerebral endothelial cells produced by their treatment with oxidized low-density-lipoproteins[32]. In addition, we found that pre-treatment of brain endothelial cells in culture with low doses of resveratrol improved their viability following oxygen glucose deprivation which could also explain improved BBB function following cerebral ischemia. Improved viability is consistent with the resveratrol-mediated protection of cerebral endothelial cells against apoptosis induced by oxidized low-density lipoproteins [33]. It is also consistent with the demonstrated protection of cardiac endothelial cells against death or injury produced by high glucose induced oxidative stress[34], [35]. In these cardiac endothelial cells, resveratrol attenuated oxidative stress that was shown to involve the activation of SIRT1 and upregulation of antioxidant defense mechanisms mediated by activation of nuclear factor-E(2)-related factor-2. We also found that resveratrol-mediated protection against oxygen glucose deprivation in endothelial cells is at least partially dependent on SIRT1 activation and in brain is associated with a reduction in NT indicative of an inhibition of nitroxidative stress. Thus improved blood brain barrier function observed in vivo is likely due to a combination of resveratrol's inhibition of MMPs, reduced degradation of tight junction proteins, reduced oxidative stress, and improved endothelial cell viability. Further study is required to demonstrate their relative importance and the extent to which protection of endothelial cells, a key component of the neurovascular unit, benefits neuronal function and viability.

To conclude, the current results indicate that a recurrent stroke consisting of two mild transient ischemic episodes produces damage exceeding that of a single mild stroke. Resveratrol administered at moderate doses and within a clinically relevant acute time frame of three days reduces parenchymal brain damage and inflammation produced by either a single or a recurrent mild stroke. These beneficial effects of resveratrol treatment appear related at least partially to a reduced BBB extravasation and cerebral edema associated with improved viability of endothelial cells against deprivation of substrate and oxygen delivery. The results support further investigation of the protective role of resveratrol treatment as a dietary supplement that could potentially provide a low risk and effective option for protective therapy in patients following their first TIA or minor stroke.

## Materials and Methods

### Ethics Statement

For experiments conducted in Calgary, animals were used and handled in accordance with the Canadian Council on Animal Care and University of Calgary Health Sciences Animal Care Committee (Approved Protocol MO7073). For laser speckle flow and blood-brain barrier (BBB) experiments conducted in Szeged, Hungary, all experimental procedures were approved by the Ethical Committee for Animal Care of the University of Szeged, Szeged, Hungary. All animals were randomized to their treatment groups and the surgeon and investigators performing analysis were blinded to the treatments.

### Bioavailability of Resveratrol

Resveratrol levels in blood and tissue were first assessed using LC/MS/MS in 200–300 g male Wistar rats (Charles River, Montreal, Canada) treated with either trans-resveratrol (25 mg/kg p.o, n = 3) or vehicle (n = 3). Blood samples were obtained in anesthetized rats at various time points (10, 20, 30, 60, 120 min) following daily treatments and the plasma was frozen for analysis. Two hours after the last (fourth) treatment, samples of brain and liver were dissected on ice, homogenized in lysis buffer, centrifuged at 5000 g for 15 min, and the supernatant was frozen. The sample preparation and analysis was as described previously with some modifications [36]. Briefly, proteins in samples (200 µL) were precipitated using 400 µL of EtOH with internal standard (resveratrol-phenyl-^13^C_6_, Sigma-Aldrich Co. Oakville, Canada). The supernatant was dried and then reconstituted in 200 µL of 50∶50 acetonitril:1 mM ammonium formate. The LC/MS/MS analysis was carried out with an Agilent 1100 HPLC (Agilent Technologies, Palo Alto, USA) coupled to an API 4000 triple quadrupole Mass Spectrometer equipped with a Turbo-Ionspray Interface (AB Sciex, Concord, Ont., Canada). Resveratrol was separated using a 2.1×100 mm, 5 µ Hypersil ODS column (Agilent Technologies, USA). The mobile phase composed of 1 mM ammonium formate (A) and acetonitrile (B) was delivered at a flow rate of 0.3 mL/min. A binary gradient started with 85% of A and 15% of B, which was then increased to 50% of B at 6 min. and subsequently to 70% of B at 8 min. Then, the column was equilibrated with 15% of B for 10 min before the next 5 µL sample injection. The mass spectrometer was operated in negative, multiple reaction monitoring mode with the following settings: source temperature 350°C, curtain gas 10, collision gas 6, gas1 50, gas2 50, and ion spray voltage −4200 V. Quantitation of trans-resveratrol (Sigma-Aldrich Co) was achieved using the 227/185 transition with the 227/143 transition monitored as confirmation ion. Transitions monitored for internal standard (resveratrol-phenyl-^13^C_6_) were 233/149 and 233/191.

### In vivo stroke model and resveratrol treatment

Thirty rats were randomized to receive either single mild (30 min transient MCAO, n = 16) or recurrent mild stroke (2×30 min transient MCAO, n = 14). Animals receiving a single stroke were further randomized to receive, daily for 3 days prior to the stroke, either vehicle (p.o., 17% Jello powder in water, n = 9) or trans-resveratrol in vehicle (25 mg/kg p.o. Sigma-Aldrich, Oakville, Ont, Canada, n = 7), the dose being within ranges used previously [5], [7], [9] (Fig.1 A). Animals designated to receive recurrent stroke were also randomized to receive resveratrol (n = 7) or vehicle (n = 7) daily for three days, but the first dose was administered 1 hour following the initial stroke and the final dose 1 hour prior to the second stroke. In ten additional animals, a lack of a significant effect of sham surgery was confirmed by comparing the injury in animals undergoing a mild ischemic stroke (n = 5) to those that underwent a sham procedure involving surgical isolation of the MCA followed by a mild ischemic stroke 3 d later (n = 5).

Transient focal ischemia was achieved by placing a microaneurysm clip (Codman, size#1) on the middle cerebral artery through a small burr hole in the skull, as previously described, with some modifications [3]. Briefly, the animal was anesthetized with isoflurane. Tympanic membrane temperature was maintained using a servo-regulated overhead heating lamp at 36.0°C, corresponding to approximately 37.0–37.5°C body temperature. The tail artery was cannulated to continuously monitor arterial blood pressure and obtain blood samples for determination of blood gas and glucose levels during the procedure. Both common carotid arteries were isolated and then an incision was made in the temporalis muscle. The skin and muscle were retracted to expose the skull. A small craniotomy was made at the point where the MCA crossed the rhinal fissure. Prior to removing the dura over the MCA, two additional burr holes were made to allow for measurement with laser Doppler flowmetry. One hole was made 3 mm dorsal to the site of the MCA (referred to as proximal) and other 3 mm posterior to the first hole (referred to as distal). The dura was removed to expose the MCA which was subsequently occluded with the microaneurysm clip. Concurrently, both common carotid arteries were occluded using vascular clamps. Blood flow reduction was confirmed with laser Doppler flowmetry. Animals were excluded if the criterion of 90% blood flow reduction in the core was not met. At the end of the 30 min occlusion, the microclip was removed and the carotid artery clamps were removed. For animals that were to undergo a second stroke, artificial dura (Gore Preclude MVP, Better Hospital Supplies Corp., Miami, FL) was placed over the exposed MCA to minimize adhesion to the vessel and fibrosis infiltration. All wounds were securely sutured and topical anesthetic was applied (Lidocaine). Analgesia (0.03 mg/kg buprenorphine s.c) was administered and animals were monitored closely during recovery. Three days later, rats to receive recurrent stroke underwent transient MCAO in a similar manner as described above. The position of the second clipping of the MCA was immediately dorsal to the original clip position to minimize trauma to the vessel. Following surgery rats were given softened food in order to minimize weight loss (<10%).

### Telemetry Measurements

To simultaneously monitor core temperature, heart rate and mean arterial blood pressure in awake and freely moving rats we used telemetry probes (TML2 C50-PXT, Transoma Medical, St Paul, MN, USA). These were implanted abdominally using aseptic techniques under isoflurane anesthesia. Two leads from the probe, tunneled under the skin, measured heart rate and a catheter inserted into the descending aorta without obstructing blood flow measured arterial blood pressure. After 1 week of recovery, animals were placed in separate cages, allowing measurements to be recorded every 30 sec via RPC-1 receivers (Transoma Medical). Following 24 h of baseline measurements, rats were fed vehicle (n = 3) or 25 mg/kg RSV (n = 3) daily for 4 doses. One hour following the final dose, rats were subjected to a single mild stroke as described above. After recovery animals were further monitored using telemetry for 48 h following stroke.

### Histological Assessment of Cell Death, Microglial Activation and Nitroxyl Radicals

One week after the either the single stroke or the 2^nd^ insult of recurrent stroke, animals were perfused with 10% formalin under anesthesia. Brains were embedded in paraffin and 6 µm sections were stained with hematoxylin and eosin to determine cell death. Standard infarct volume assessment did not allow us to accurately evaluate the scattered cell death that we observed following mild stroke; therefore, a cumulative score for brain injury at each level was assessed by an investigator blinded to the animal identity. A scoring system similar to that used previously [3] was used to determine the extent of injury. The cortical hemisphere was divided into 4 areas of interest that were scored from 0–4 as follows: 0 for normal, 1 for <10% of neuronal injury, 2 for 10–50% neuronal injury, 3 for >50% neuronal injury and 4 for confluent areas of pan necrosis.

Immunohistochemical staining was performed in adjacent sections that were dewaxed and incubated first with 10% goat serum, then primary antibody overnight, and then visualized using the appropriate biotin-conjugated IgG (Jackson ImmunoResearch Lab followed by incubation with peroxidase-congugated streptavidin (HRP) (1∶400, Dako) and diaminobenzidine (DAB, Sigma). Reactive astrocytes were assessed using anti-glial fibrillary acidic protein (rabbit anti-GFAP, 1∶1000, Sigma). Inflammatory signs of activated microglia/macrophages were assessed using anti-ED1 (mouse anti-rat CD68 clone ED1, 1∶100, Serotec). Signs of oxidative stress were assessed using rabbit anti-Nitrotyrosine (NT, 1∶1000, Upstate Biotechnology, NY)) to detect the accumulation of peroxynitrite metabolites indicative of damage related to reactions with nitroxyl radicals. Antigen retrieval was performed prior to incubation with primary antibodies by immersing slides in boiling citrate buffer pH = 6 for 10 mins for ED1 and NT.

Areas of altered staining were measured in GFAP stained sections that were scanned digitally (Nikon COOLSCAN 5.0). Areas of altered stain were corrected for edema by measuring the contralateral area and the unaltered area of staining ipsilaterally and presenting the difference as a percentage of the contralateral area. The ED1 and NT sections were scanned and assigned scores similar to the hematoxylin and eosin stained sections above, except that increased levels of staining were scored in the four different cortical areas as follows: 0 - no increase in staining, 1- modest increase in <25% of the area, 2 - modest increase in 25–75 of the area and/or intense increase in <25% of the area, 3 – modest increase in >75% and/or intense increase in 25–75% and 4 – intense increase in >75% of the area.

### Laser Speckle Contrast Imaging and Analysis

Acute regional CBF with the production of the second stroke was measured using laser speckle imaging in another group of 13 rats. Animals were exposed to recurrent stroke and treated with either resveratrol (n = 7) or vehicle (n = 6), for the 3 days between the first and second stroke. After carotid artery isolation, rats were placed into a standard stereotaxic frame and the skull was exposed with a midline scalp incision. The ipsilateral parietal bone was carefully thinned with a water-cooled drill (Bien-Air, Sweden) and covered with a UV-light cured doming (Permabond, UK) to render the skull transparent. A lateral window was also prepared to expose the distal MCA for clipping. Rats were placed under a PeriCam PSI HD System (Perimed, Sweden), which utilizes a built-in 70 mW laser diode to illuminate the cortical surface. Blood perfusion in the scanned area was monitored using a 1388×1038 pixel CCD camera at a speed of 2 Hz. Ten minutes of baseline was recorded, after which the 2^nd^ 30 minutes transient MCAO was performed as described above. Images were taken (2 per sec) during the entire ischemic period and for 1 hour of reperfusion. Analysis of laser speckle images was performed using PIMsoft; a PeriCam PSI dedicated software (Perimed, Sweden). Maps of the speckle contrast value at each pixel of the image show the pattern of blood flow on the cortical surface during imaging. The mean perfusion of the parenchymal microcirculation was calculated by measuring the average perfusion values in four regions of interest of equal size and shape placed in four conserved locations (labeled 1–4 in Fig. 4 A) within the ipsilateral cortex.

### Measurement of BBB Permeability and Water Content

BBB integrity and water content were investigated in the same animals as those used for measuring CBF changes. Blood brain barrier permeability was assessed by measuring extravasated Evans Blue dye in animals following exposure to recurrent stroke and treated with either resveratrol (n = 7) or vehicle (n = 6). For treatment, 4 ml/kg of 2% Evans Blue (Sigma, USA) in saline was injected through the tail artery immediately prior to reperfusion with the recurrent stroke. Rats were sacrificed 48 hours after reperfusion by transcardial infusion of saline. Wet and dry weights (dried at 60°C for 24 h) of the ipsilateral and contralateral cerebral cortices were measured and the corresponding water content was calculated by the formula: water content = 100% (wet weight–dry weight)/wet weight.

Dry cortices were incubated overnight in formamide (5 ml/g dry weight) and the amount of Evans Blue released into the formamide was quantified by florescence spectrophotometry (excitation 620 nm; emission 680 nm). Raw data were compared with a standardized curve of known quantities of Evans Blue in formamide. Results are presented in ng/g tissue.

### Rat Brain Endothelial Cell Cultures

Plastics used for endothelial cultures were obtained from Becton-Dickinson (BD Biosciences, San Jose, CA, USA). Dulbecco's modified Eagle medium (DMEM), Neurobasal medium, B27 Supplement, 2-mercaptoethanol, and fetal bovine serum were obtained from Invitrogen (Carlsbad, CA, USA) and fetal bovine plasma-derived serum was purchased from Animal Technologies (Tyler, TX). Dispase I was purchased from Roche (Mannheim, Germany) and Collagenase type II was obtained from Worthington (Lakewood, NJ). CellTiter 96 AQ_ueous_ One Solution Assay was acquired from Promega (Madison, WI, USA).

A total of 3 Wistar dams and 22 pups were used for endothelial cultures. Rat brain endothelial cells were isolated from two week old Wistar rat pups (Charles River Laboratories [Montreal, Canada]), as described previously [37], [38]. The cortices were separated from meninges, then homogenized, and digested. The homogenate was mixed with 20% BSA and centrifuged at 1,000 g for 20 min to isolate cortical microvessels. Microvessels were washed in DMEM, further digested, layered on a continuous 33% Percoll gradient, and centrifuged at 1,000 g for 10 min. Endothelial cells formed an easily distinguishable band that was aspirated and washed twice with DMEM. The cells were then seeded onto collagen IV- and fibronectin-coated 96-well plates. Endothelial cell growth medium consisted of DMEM supplemented with 2 mM glutamine, 20% fetal bovine plasma-derived serum, 50 µg/ml endothelial cell growth supplement, 100 µg/ml heparin, 1 ng/ml basic fibroblast growth factor, 5 µg/ml vitamin C, and various antibiotics. Purity of endothelial cell cultures was ensured by treating cells with puromycin (3 µg/ml) for three days as described and validated by Perriere et al. [39] and was also confirmed for each plate by visual inspection of cell morphology. Experiments were carried out on days 3–7 in vitro.

An ischemic type insult sufficient to evoke ∼50% cell death was produced using oxyygen and glucose deprivation by washing the cells and replacing the medium with glucose-free Dulbecco's modified Eagle medium (DMEM). This was followed by exposure of the cells to a humidified anaerobic environment for 3.5 hours. Control cell cultures were incubated under normal conditions in glucose-containing DMEM.

Cell viability in the cultures was determined 24 hours after oxygen glucose deprivation using the tetrazolium-based CellTiter 96 AQueous One Solution assay. Absorbance at 490 nm was measured with a microplate reader (FLUOstar OPTIMA, BMG Labtech GmbH, Offenburg, Germany). Comparisons were made between cultures treated in a similar way, with the exception of omitting the noxious stimulus. Cell viability (%) was expressed as: (absorbance_sample_−absorbance_background_×100/(absorbance_control_−absorbance_background_).

Resveratrol's effects were investigated in cell cultures treated with various doses of resveratrol (100 nM-10 µM) dissolved in culture medium containing up to 1.5% DMSO. Control cultures were treated with 1.5% DMSO alone. Cells were treated once a day (a new resveratrol solution was made each day) for three consecutive days to match the *in vivo* treatment paradigm, and the medium was replaced with each new treatment. Alternatively, cells were co-treated with resveratrol and 10 µM or 50 µM sirtuin-1 (SIRT1) inhibitor (sirtinol, Sigma) or the non-specific nitric oxide synthase blocker (N_ω_-Nitro-L-arginine methyl ester hydrochloride (L-NAME, Sigma).

### Statistical Analysis

Differences between treatments were assessed with t-test or a one-way analysis of variance (ANOVA) and a Student–Newman–Keuls test. Non-parametric data were analyzed using a Kruskal Wallis ANOVA on ranks and a Mann–Whitney rank sum test. A value of P<0.05 was considered significant.
